# QbD Design, Formulation, Optimization and Evaluation of Trans-Tympanic Reverse Gelatination Gel of Norfloxacin: Investigating Gene-Gene Interactions to Enhance Therapeutic Efficacy

**DOI:** 10.3390/gels9080657

**Published:** 2023-08-15

**Authors:** Amit Budhori, Abhishek Tiwari, Varsha Tiwari, Ajay Sharma, Manish Kumar, Girendra Gautam, Tarun Virmani, Girish Kumar, Abdulsalam Alhalmi, Omar Mohammed Noman, Sidgi Hasson, Ramzi A. Mothana

**Affiliations:** 1 Devsthali Vidyapeeth Institute of Pharmacy, Lalpur, Rudrapur 263148, India; budhori.amit@gmail.com; 2 Pharmacy Academy, IFTM University, Moradabad 244102, India; 3 School of Pharmaceutical Sciences, Delhi Pharmaceutical Sciences and Research University, Pushp Vihar, New Delhi 110017, India; ajaysharmapharma1979@gmail.com; 4 School of Pharmaceutical Sciences, CT University, Ludhiana 142024, India; manish_singh17@rediffmail.com; 5 Shri Ram College of Pharmacy, Muzaffarnagar 251001, India; dr.girendra@gmail.com; 6 School of Pharmaceutical Sciences, MVN University, Palwal 121105, India; tarun.virmani@mvn.edu.in (T.V.); girish.kumar@mvn.edu.in (G.K.); 7 Department of Pharmaceutics, School of Pharmaceutical Education and Research, Jamia Hamdard, New Delhi 110062, India; asalamahmed5@gmail.com; 8 Department of Pharmacognosy, College of Pharmacy, King Saud University, P.O. Box 2457, Riyadh 11451, Saudi Arabia; onoman@ksu.edu.sa (O.M.N.); rmothana@ksu.edu.sa (R.A.M.); 9 School of Pharmacy and Biomolecular Sciences, Liverpool John Moores University, Liverpool L3 5UG, UK; s.s.hasson@ljmu.ac.uk

**Keywords:** reverse gelatination gels, norfloxacin, controlled drug delivery, ear infections, Quality by Design

## Abstract

Traditional otic drug delivery methods lack controlled release capabilities, making reverse gelatination gels a promising alternative. Reverse gelatination gels are colloidal systems that transition from a sol to a gel phase at the target site, providing controlled drug release over an extended period. Thermosensitive norfloxacin reverse gelatination gels were developed using a Quality by Design (QbD)-based optimization approach. The formulations were evaluated for their in vitro release profile, rheological behavior, visual appearance, pH, gelling time, and sol–gel transition temperature. The results show that the gelation temperatures of the formulations ranged from 33 to 37 °C, with gelling durations between 35 and 90 s. The drug content in the formulations was uniform, with entrapment efficiency ranging from 55% to 95%. Among the formulations, F10 exhibited the most favorable properties and was selected for a stability study lasting 60 days. Ex-vivo release data demonstrate that the F10 formulation achieved 95.6percentage of drug release at 360 min. This study successfully developed thermosensitive norfloxacin reverse gelatination gels using a QbD-based optimization approach. The selected formulation, F10, exhibited desirable properties in terms of gelling temperature, drug content, and release profile. These gels hold potential for the controlled delivery of norfloxacin in the treatment of ear infections.

## 1. Introduction

Otitis media is a common type of ear infection that primarily affects the middle ear, which is the space behind the eardrum [1]. It occurs when the Eustachian tube, which connects the middle ear to the back of the throat, becomes blocked or infected. Otitis media can be caused by bacterial or viral infections, and it is more prevalent in children, although it can also affect adults [2]. The condition often presents with symptoms such as ear pain, hearing loss, fluid drainage from the ear, and sometimes fever [3]. Prompt diagnosis and appropriate treatment are important to alleviate symptoms, prevent complications, and preserve hearing function [4]. Treatment options may include antibiotics, pain relievers, and in some cases, surgical intervention to address persistent or recurrent infections [5]. While various treatment options exist, the development of novel drug delivery systems holds promise for enhancing therapeutic efficacy and patient compliance.

In recent years, the field of pharmaceutical research has witnessed significant advancements in the integration of Quality by Design (QbD) principles, and innovative formulation strategies [6]. These multidisciplinary approaches aim to optimize drug delivery and improve therapeutic outcomes [7]. This article presents a comprehensive approach to address ear infections by employing the principles of QbD design, and the formulation of a novel trans-tympanic reverse gelatination gel of norfloxacin. The objective is to develop an efficient and patient-friendly drug delivery system that enhances drug penetration, bioavailability, and residence time in the middle ear.

The formulation of a trans-tympanic reverse gelatination gel provides a unique approach to drug delivery, specifically targeting the middle ear [8]. This innovative gel system undergoes a transformation from a liquid to a gel state upon contact with body temperature, allowing for prolonged drug release and enhanced residence time at the site of infection. The integrity of the tympanic membrane plays a crucial role in facilitating the delivery of drugs to the middle and inner ear [9]. The effectiveness of transtympanic administration relies on the diffusion of drugs through the intact tympanic membrane and into the middle and inner ear. To expedite this process, permeation boosters are employed. Through the utilization of chemical permeation enhancers within a hydrogel, studies have shown that antibiotics can be directly delivered to the middle ear to treat otitis media [10,11].

Polymer-based reverse gelation gelling solutions are liquid formulations that exhibit high viscosity and mucoadhesive properties, and are composed of polymers [11,12,13]. They can transform from a liquid to a gel state in the ear canal due to changes in temperature and pH [14]. By adjusting the residence time of the drug in the ear, the amount delivered can be effectively controlled, thereby preventing ear disorders [15]. Reverse gelation gels, being sensitive to temperature and pH, can be administered orally, ocularly, rectally, vaginally, or via injection. They can be formulated using either natural or synthetic polymers [15,16]. These in situ-forming gel systems have a wide range of medicinal applications, including drug delivery, tissue healing, and cell encapsulation [17,18]. The process of reverse gelation and gel formation can be achieved through various techniques such as solvent exchange, UV irradiation, ionic cross-linking, pH adjustment, and temperature modulation [19,20,21,22,23].

In the case of otic gels formed through reverse gelation, they offer several advantages. These include longer durations of action, which improves patient compliance and comfort, lower drug dosage requirements, reduced frequency of administration, and the ability to be packaged in standard droppers and containers used for solutions [24,25]. This makes them a unique drug delivery system for the noninvasive delivery of antibiotics and gene therapy vectors to the middle ear. This article aims to contribute to the growing body of knowledge in this field, providing a comprehensive understanding of the development and evaluation of the trans-tympanic reverse gelatination gel of norfloxacin as a potential therapeutic option for ear infections.

## 2. Results and Discussion

### 2.1. Response Surface Analysis by Design Expert

#### 2.1.1. Percentage Entrapment Efficiency

**Figure 1:**
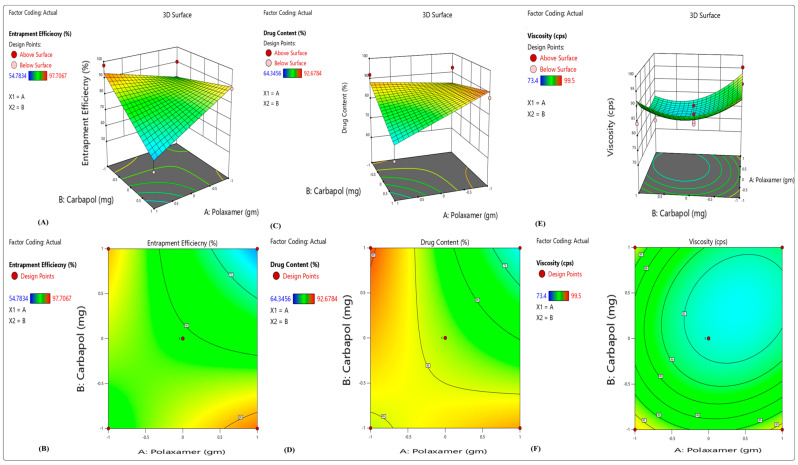
3D response surface plots showing the effects of independent factors on (**A**,**B**) percentage entrapment efficiency, (**C**,**D**) percentage drug content and (**E**,**F**) viscosity.

There were wide variations in the results for EE, with a mean of 73.49 percent and a maximum of 95.78 percent (F10) depending on the variable level selected (Figure 1). The %EE of polynomial equation is shown below:

Y_1_ (% Entrapment efficacy) = 74.28147 + 17.89000X_1_ − 10.22000X_2_ + 6.66000X_3_ + 19.20000X_1_X_2_ − 6.90000X_1_X_3_ − 0.32000X_2_X_3_ (1)

From the above polynomial equation, we can see that the variables Polaxamer-407 (X_1_) and HPMC K100 (X_3_) had a positive effect on the EE, whereas increasing the concentration of carbopol-940 (X_2_) causes a decrease in EE. Higher emulsifier-to-lipid ratios may lead to an increase in EE; this might be because there was enough emulsifier to keep norfloxacin in the lipid particles or on their surface [26].

#### 2.1.2. Percentage Drug Content

From 75.18 percent (F9) to 85.9 percent (F10), a total of 80.28 percent was issued in total (Figure 1). The following polynomial equation shows the impacts of many independent factors on the release of medication:

Y_2_ (percentage of drug release) = 71.68 + 17.52000X_1_ − 11.090000X_2_ − 4.53000X_3_ − 6.82000X_1_X_2_ − 0.80000 X_1_X_3_ + 3.70000X_2_X_3_ (2)

From the above polynomial equation, we see that the variable Polaxamer-407 (X_1_) had a positive effect on the percentage of drug release, whereas by increasing the concentration of carbopol-940 (X_2_) and HPMC K100 (X_3_), there is a decrease in percentage of drug release. As a possible explanation, it might be that the carriers have more molecules of drugs on their surfaces. Polymer or drug ratio determines the particle size, and the larger the particle, the lower the percentage release of the drug. The significance and amount of interaction between independent and dependent variables were determined using a two-way ANOVA. For analyzing the interactions between the independent variables, a 3D surface was generated using the regression model [27].

#### 2.1.3. Viscosity

Viscosity (Y_3_) ranged from 1427.56 cps to 1621.85 cps in the replies (7). Viscosity was shown to be inversely related to drug release in vitro. Coded values of factor levels were used in the response models to assess the quantitative effects of the different combinations and levels of variables on drug release and viscosity. Full model equations might be used to express the model in question:

Y_3_ (Viscosity) = 1542.56 + 26.16100X_1_ + 1467.66700X_2_ − 1.75000X_3_ − 18.30000X_1_X_2_ + 1.20000 X_1_X_3_ − 0.50000X_2_X_3_ (3)

From the above polynomial equation, we see that the variables Polaxamer-407 (X_1_) and carbopol-940 (X_2_) had a positive effect on the viscosity, whereas by increasing the concentration of HPMC K100 (X_3_), a decrease in viscosity results. The results of the ANOVA test show that a polynomial model with a negative sign indicated a negative effect was the most appropriate. Microemulsion-based topical gel drug release was negatively impacted by the addition of X_1_ and X_2_ to the simplified model equation. The equation leads to this result. When X_1_ and X2 (poloxamer 407 concentration) are raised, the medication’s percentage release decreases. Viscosity was raised when the Polaxamer 407 concentration was increased. As a result, the molecules of drug faced a higher barrier to diffusion.

### 2.2. UV Spectroscopy and Compatibility Study

The UV spectroscopy and calibration of norfloxacin were included in the supplementary file. Figure S1 shows the calibration curve of norfloxacin in methanol, distilled water and phosphate buffer at pH 7.4. The results of IR compatibility studies are also included in the supplementary file. Figure S2 shows the IR spectra of norfloxacin and Polaxamer 407, norfloxacin and carbapol-940, and norfloxacin and HPMC. The IR compatibility study of norfloxacin with poloxamer 407, carbopol 940, and HPMC reveals no significant chemical interactions. The components maintain their original molecular structures, ensuring the stability and integrity of the formulation. This information is crucial for establishing the compatibility of the excipients with norfloxacin in the development of a successful and effective pharmaceutical product.

### 2.3. Test for Appearance/Clarity

White and dark were used to visually inspect the produced mixtures. All formulations were discovered to be clear and transparent.

### 2.4. Sol–Gel Transition Temperature

The temperature for otic gel formation, also known as the sol–gel transition temperature, typically ranges between 30 °C to 40 °C. This temperature range is selected to ensure that the otic gel remains in a liquid state at room temperature and during administration. Once the gel is placed in the ear canal, body heat (which is around 37 °C) triggers the gelation process, causing the formulation to change from a liquid to a gel. The specific temperature within this range may vary depending on the formulation and the intended application. It is important to choose a sol–gel transition temperature that is slightly above body temperature (37 °C) to ensure that the gel forms and adheres effectively to the ear canal, allowing for prolonged drug release and therapeutic effect.

The sol–gel transition temperatures of the formulations were found to range from 33 ± 0.68 °C to 36 ± 0.76 °C (Table 1). This indicates that the formulations would undergo gelation at temperatures slightly above these values. Selecting an appropriate sol–gel transition temperature is vital to ensure the gel forms at the intended application site, such as the ear. The transition temperature should be slightly above body temperature, allowing the formulation to gel effectively and adhere to the target area for prolonged drug release and better therapeutic outcomes. Based on the study results, formulation F10 exhibited the lowest sol–gel transition temperature among all the formulations, making it a potentially suitable candidate for in situ gelling in the ear. The lower transition temperature of F10 suggests that it would undergo gelation at a temperature closer to body temperature, enhancing its potential for targeted drug delivery and therapeutic efficacy. The gelling time of each formulation is the time taken for the liquid formulation to transform into a gel after reaching the sol–gel transition temperature. The gelling time ranged from 35 ± 0.34 s to 120 ± 0.39 s for different formulations. This parameter is crucial for understanding how quickly the formulations change their physical state upon reaching the sol–gel transition temperature. For instance, formulation F10 would gel at a temperature of approximately 33.68 °C. Formulation F10 with the lowest transition temperature appears promising for targeted drug delivery. The sol–gel transition temperature is a crucial property of in situ gelling systems, as it determines the temperature at which the formulation changes from a liquid to a gel.

### 2.5. Rheological Investigations

**Figure 2:**
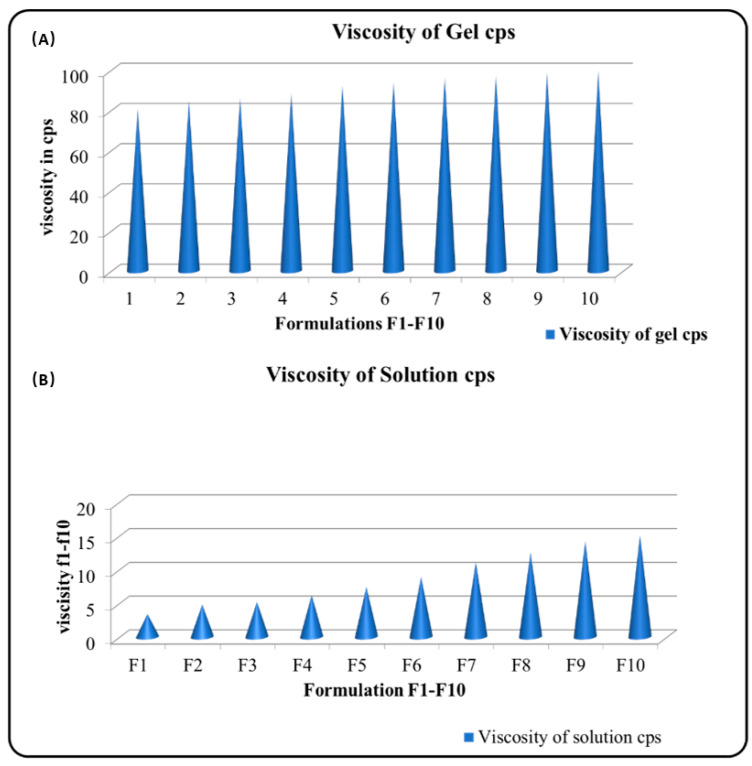
Viscosity of (**A**) gel and (**B**) solutions.

Table 2 and Figure 2 (viscosity of gel and solution) illustrate the results of the rheological investigation. The viscosity of the formulations in solution state is relatively low, ranging from 80.3 cp to 96.5 cp. This suggests that the formulations are fluid and easy to apply. The viscosity of the formulations in gel state is significantly higher, ranging from 1427.56 cp to 1621.85 cp. This suggests that the formulations are more viscous and will stay on the skin for longer periods of time.

The spreadability of the formulations is also variable, ranging from 5.46 to 6.35. This suggests that some formulations are easier to spread than others. The formulations with higher spreadability may be easier to apply, but they may not be as effective at delivering the drug to the ear. The gel strength of the formulations is also variable, ranging from 10.65 to 65.78. This suggests that some formulations are more effective at holding their shape than others. The formulations with stronger gel strength are more likely to hold their shape, which could be important for delivering the drug to the ear.

The viscosity of the formulations is likely due to the composition of the formulations. The formulations contain a variety of gelling agents, which can affect their viscosity of the formulations.

The spreadability of the formulations is likely due to their viscosity as well as their surface tension. Formulations with lower viscosity and lower surface tension are more likely to spread easily.

The gel strength of the formulations is likely due to the interactions between the gelling agents and the other components of the formulations. The formulations with stronger gel strength are more likely to hold their shape.

The best formulation for otic gel depends on a number of factors, including the drug being delivered, the target area, and the patient’s individual needs. However, based on the data provided, formulation F10 appears to be a good candidate for further development. Formulation F10 has a relatively high viscosity in the gel state, which could help it stay in the ear for longer periods of time. It also has a relatively high spreadability, which could make it easier to apply. Additionally, F10 has a strong gel strength, which could help it hold its shape and deliver the drug to the ear effectively.

### 2.6. pH of Formulation Determination

All the formulations produced in this study had pH values ranging from 6 to 7.4, which are within the normal range for otic gels (Table 3). The pH stability of each formulation was assessed over a 48 h period. Formulation F1 showed a significant decrease in pH after 24 h, but then the pH increased again after 48 h, indicating instability. Formulation F2 exhibited a significant decrease in pH after 24 h, which continued to decrease after 48 h, suggesting excessive acidity and potential safety concerns for ear use. In contrast, formulations F3, F4, F6, F7, F8, and F9 maintained relatively constant pH levels over time, indicating stability and safety for ear application. Formulation F5 showed a significant increase in pH after 24 h, followed by a decrease after 48 h, suggesting pH instability may be caused by ear interactions. Formulation F10 consistently maintained a slightly higher pH compared to other formulations. This slight pH elevation may be attributed to the buffering capacity of the formulation components. Notably, the pH of formulation F10 remained stable over time, indicating good stability and safety for ear use. All formulations were deemed safe for ear application, but variations in pH stability were observed. Formulation F3 exhibited the highest stability, while formulation F2 showed the lowest stability. Considering its stability, safe pH range, and slightly higher pH, formulation F10 emerges as a promising candidate for further development in treating ear infections.

### 2.7. Zeta Potential

**Figure 3:**
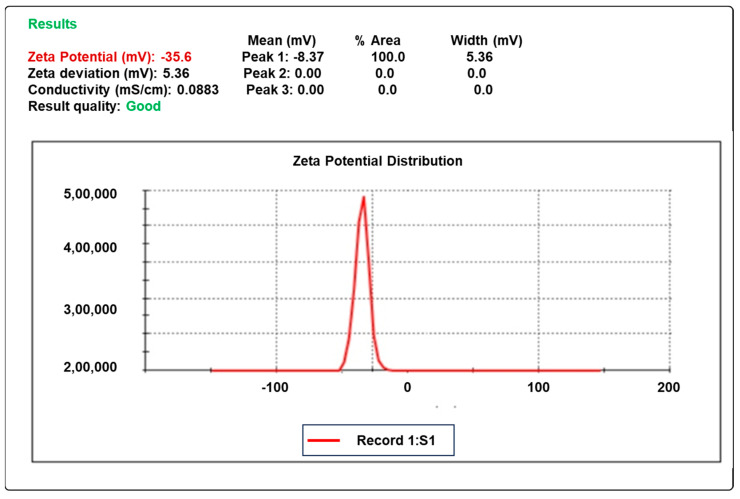
The zeta potential of the optimized formula F10.

The zeta potential of the dispersion determines the stability of colloidal dispersion. The optimal zeta potential values, which indicated stability and did not create aggregates, were found to be in the vicinity of −35.6 mV, as shown in Figure 3.

### 2.8. Release Kintics

Zero-order, first-order, and Higuchi models have been used in Figure S3 and Table 4 to characterize the release kinetics. The release kinetics of norfloxacin from the formulations were evaluated using different mathematical models. The zero-order model, first-order model, Higuchi model, Hixson–Crowell model, and Korsmeyer–Peppas model were all used to fit the release data. The results show that the Korsmeyer–Peppas model was the best fit model for all of the formulations. This model is a more complex model than the other models, and it takes into account the diffusion and erosion of the drug from the formulation. The Korsmeyer–Peppas model is also able to fit the data over a wider range of release times than the other models. The release rate constants for the formulations ranged from 15.46 to 23.38 µg/h. The formulation with the highest release rate was F10, and the formulation with the lowest release rate was F2. The release kinetic findings suggest that the formulations release norfloxacin by a combination of diffusion and erosion. The Korsmeyer–Peppas model is able to describe this complex release mechanism, and it is therefore the best-fitting model for the data. The release rate constants for the formulations can be used to predict the release profiles of the formulations. The formulations with higher release rate constants will release norfloxacin more quickly than the formulations with lower release rate constants.

### 2.9. Drug Content and Entrapment Efficiency

Table 5 shows the drug content and entrapment efficiency of different formulations of norfloxacin. The drug content is the percentage of norfloxacin in the formulation, and the entrapment efficiency is the percentage of norfloxacin that is entrapped in the formulation. The results show that the drug content of the formulations ranged from 75.18% to 85.9%. The entrapment efficiency of the formulations ranged from 52.10% to 95.78%. Formulation F10 had the highest drug content and entrapment efficiency. The drug content of formulation F10 was 85.9%, and the entrapment efficiency was 95.78%. This suggests that formulation F10 is the most effective formulation for delivering norfloxacin. Formulation F2 had the lowest drug content and entrapment efficiency. The drug content of formulation F2 was 83.11%, and the entrapment efficiency was 55.98%. This suggests that formulation F2 is the least effective formulation for delivering norfloxacin. The findings suggest that the drug content and entrapment efficiency of the formulations are important factors for determining the efficacy of norfloxacin therapy. The formulations with higher drug content and entrapment efficiency are likely to be more effective in delivering norfloxacin to the target site.

### 2.10. Cumulative Drug Release

**Figure 4:**
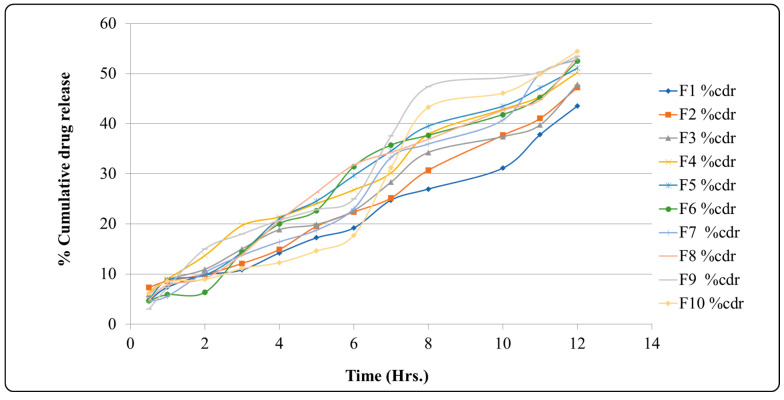
Graph of percentage of cumulative drug release.

Table 6 and Figure 4 show the percentage cumulative drug release (%CDR) of pure norfloxacin and the optimized formulation (F10) at different time points. The results show that the %CDR of the optimized formulation is consistently higher than the %CDR of the pure drug. At 0.5 h, the %CDR of the pure drug is 6.22%, while the %CDR of the optimized formulation is 0.39%. This suggests that the optimized formulation releases norfloxacin more quickly than the pure drug. At 1 h, the %CDR of the pure drug is 8.55%, while the %CDR of the optimized formulation is 5.15%. This suggests that the optimized formulation continues to release norfloxacin more quickly than the pure drug over time. At 12 h, the %CDR of the pure drug is 54.44%, while the %CDR of the optimized formulation is 81.29%. This suggests that the optimized formulation is able to release a significantly higher percentage of norfloxacin than the pure drug over a 12 h period. The findings suggest that the optimized formulation is a more effective delivery system for norfloxacin than the pure drug. The optimized formulation is able to release norfloxacin more quickly and at a higher percentage than the pure drug. This suggests that the optimized formulation could be used to improve the efficacy of norfloxacin therapy.

### 2.11. Ex Vivo Permeation Study

The results are shown in Table 7. Permeation is greatest in formulation F10. The study investigated the ex vivo release profiles of 10 different formulations of an otic gel. The formulations were evaluated over a period of 360 min, and the release of the drug was measured at 15, 30, 45, 60, 75, 90, 120, 180, and 360 min. The results show that the release of the drug from the formulations was not constant over time. After a rapid initial release, the release rate slowed down. The formulations with the highest initial release rates were F1, F2, and F3. However, these formulations also had the lowest sustained release rates. The formulation with the best overall release profile was F10. This formulation had a higher initial release rate than the other formulations, then a higher sustained release rate. F10 also had the highest cumulative release of the drug at 360 min. The results of this study suggest that formulation F10 is the most promising formulation for the delivery of the drug in an otic gel. F10 has a high initial release rate, which is important for providing the rapid relief of symptoms. It also has a sustained release rate that is higher than that of the other formulations, which is important for providing long-term relief. The ex vivo release data reveal a correlation between drug release and the pH of the surrounding medium. The drug exhibited higher release rates in acidic conditions and lower release rates in basic conditions. This can be attributed to the drug’s increased solubility in acidic environments. Consequently, formulators must consider the pH of the medium when designing otic gel formulations. The findings also highlight the importance of pH in drug release kinetics, emphasizing the need for precise formulation design to achieve the desired therapeutic effect. Researchers and developers in this field can benefit from the insights gained in this study when working on future otic gel formulations.

### 2.12. Stability Studies

The results of the stability experiments are shown in Table 8. The stability studies on formulation F10 show that the formulation was stable over a period of 60 days. The pH of the formulation remained within the acceptable range of 6.5–7.5 throughout the study period. The gelling capacity of the formulation was also maintained, as indicated by the +++ rating at all time points. The viscosity of the sol (liquid) phase of the formulation increased slightly over time, from 15.1 cp at 30 days to 15.3 cp at 45 days and 14.6 cp at 60 days. The viscosity of the gel phase of the formulation remained relatively constant, with a value of 99.5 cp at all time points. These findings suggest that formulation F10 is stable over a period of 60 days under the storage conditions used in this study. The slight increase in the viscosity of the sol phase of the formulation may be due to the gradual degradation of the excipients over time. However, this increase is not significant and is unlikely to have any impact on the efficacy or safety of the formulation. Further studies are needed to evaluate the long-term stability of the formulation and assess its efficacy and safety in vivo.

### 2.13. Gene–Gene Interactions

Network pharmacology is a discipline that explores the complex interactions between drugs, target proteins, and biological pathways at a systems level. The network pharmacology analysis in this study helps identify potential targets and mechanisms of action for nofloxacin against antibiotic-resistant bacteria. It provides a holistic view of the drug’s interactions with specific genes and target proteins, shedding light on its potential impact as an effective therapeutic agent in the battle against multidrug-resistant bacteria.

**Figure 5:**
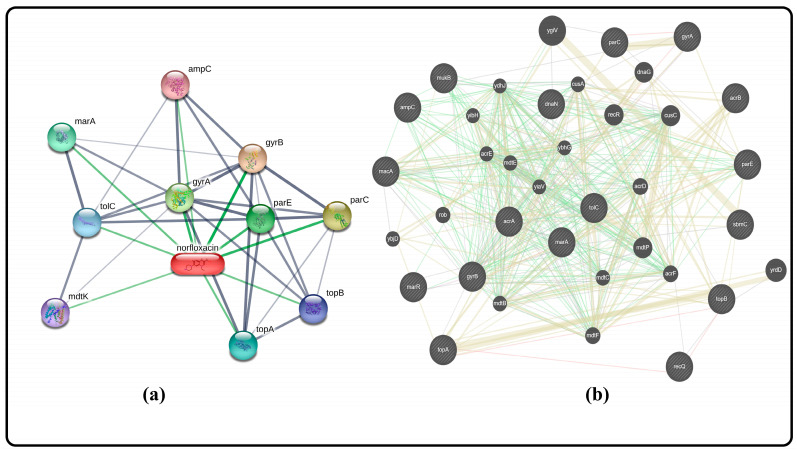
Gene–gene interaction of norfloxacin (**a**) through STITCH software gene prediction, and (**b**) through GeneMania software gene prediction.

Figure 5 shows a network pharmacology analysis that gives valuable information about the important interactions between nofloxacin and key genes that are involved in antibiotic resistance. This analysis shows that nofloxacin interacts with many important genes, such as topA, topB, mukB, recQ, dnaN, parC, parE, marA, marR, gyrA, gyrB, macA, ampC, acrA, acrB, tolC, and sbmC. Of particular importance is the AcrAB-TolC efflux pump, which plays a pivotal role in the transport of diverse chemicals, including antibiotics. The AcrAB-TolC efflux pump contributes to antibiotic resistance by actively transporting these compounds out of bacterial cells. One of the main ways that nofloxacin works is by stopping the AcrAB-TolC efflux pump from working. This stops bacteria from being resistant to the drug. By hindering the pump’s activity, nofloxacin prevents the removal of antibiotics from the bacterial cell, increasing the drug’s effectiveness in combating infections caused by multidrug-resistant bacteria. Additionally, nofloxacin also targets the gyrase subunits, gyrA, and gyrB. These subunits are essential components of the DNA gyrase enzyme, and plays a vital role in DNA replication and repair. By inhibiting gyrase, nofloxacin interferes with bacterial DNA synthesis and repair, further contributing to overcoming resistance mechanisms [28,29].

### 2.14. Discussion

The sol–gel transition temperatures of the formulations were found to be between 36 ± 0.76 and 33 ± 0.68 °C, with gelling times ranging from 35 ± 0.34 to 120 ± 0.39 s. The ability of the formulation to gel increases to some extent with temperature, but further increases in temperature lead to the collapse of the gel structure. Another important factor in the evaluation of thermosensitive in situ gel formulations is the gelling time. Reduced gelling time of the formulations is necessary for improved gelling ability. The results show that even when the concentration of the viscosity modifier is increased, Carbapol-940 and HPMC K-100 combined fail to form a gel below a specific concentration of poloxamer 407. The improved formulation has gel strengths between 10.65 ± 0.67 and 650.78 ± 0.45 cm. A strong enough gel is necessary to prevent the formulation from leaking from the ear. The gel strength of the formulation increases as the polymer concentration increases. The in-situ gel bases showed shear thinning behavior. The viscosity increased with the concentration of the viscosity-increasing agent. The viscosities of the formulations ranged from 1427.56 ± 0.39 to 1621.85 ± 3.68 cp. The norfloxacin in situ gelling system contained a fixed amount of 0.54 gm. An ex vivo drug release study was conducted for all of the selected formulations. The drug release profile for all batches was sustained for 6 h. The results show that formulation F10 had good in vitro drug release. The linear regression coefficient for each kinetic model was determined, and the pattern of drug release from the dose was predicted. It was found that the improved formulation F10 follows the Higuchi model mechanism and first-order kinetics. For the stability studies, the optimal formulation F10 was selected and stored at 4 °C in the refrigerator for 60 days. According to the evaluation, the parameters did not significantly change during the storage time. In conclusion, the results of this study show that the optimized formulation F10 has good in vitro drug release and stability. This formulation could be a promising candidate for the development of a norfloxacin in situ gelling system.

## 3. Conclusions

The results of this study show that formulation F10 has good in vitro drug release and stability. This formulation could be a promising candidate for the development of a norfloxacin in situ gelling system for the management of otitis media. However, further studies are needed to evaluate the efficacy of this formulation in vivo. In vivo studies should be conducted to assess the drug’s safety and efficacy in a relevant animal model. Additionally, the formulation should be evaluated for its potential to inhibit the growth of bacteria that are resistant to norfloxacin. The gene interaction studies show that formulation F10 inhibits the AcrAB-TolC efflux pump, gyrA, and gyrB. These are genes that are involved in antibiotic resistance. The inhibition of these genes could contribute to the efficacy of formulation F10 in treating otitis media. Overall, the results of this study are promising and suggest that formulation F10 could be a valuable therapy for otitis media. Further studies are needed to confirm these findings and evaluate the safety and efficacy of this formulation in vivo.

## 4. Materials and Methods

### 4.1. Chemicals

The chemicals required in this study are poloxamers—HPMC K100, Carbapol 940, Propyl paraben, methanol, phosphate buffer, N-octanol, glacial acetic acid, etc.—and were purchased from Merck and Loba, India.

### 4.2. Selection of Polymer

The formulation of the norfloxacin in situ gel involves the careful selection of polymers to achieve desired properties and drug delivery characteristics. Three key polymers, namely, poloxamer-407, carbopol-940, and hydroxypropyl methyl cellulose (HPMC), were chosen for their specific roles in the gel formulation. Each polymer contributes unique properties, allowing for the successful development of an effective norfloxacin in situ gel. (a) Poloxamer-407 is a thermosensitive polymer that undergoes a sol–gel transition in response to temperature changes. At lower temperatures, poloxamer-407 remains in a liquid state, facilitating ease of administration and application. However, upon contact with body heat or affected areas, it undergoes rapid gelation, transforming into a semi-solid gel form. This temperature-triggered gelation is particularly advantageous for the norfloxacin gel, as it provides a favorable solution for topical application, and once applied, it transforms into a stable gel, promoting prolonged contact time with the skin or mucosal surfaces. The gelation behavior of poloxamer-407 eliminates the need for additional chemical cross-linkers, simplifying the formulation process and minimizing potential toxicity concerns. The inclusion of poloxamer-407 is essential to providing an easy-to-administer and well-adhering norfloxacin gel product. (b) Carbopol-940, also known as carbomer, is a high-molecular-weight synthetic polymer with excellent thickening and gel-forming properties. In the norfloxacin gel formulation, carbopol-940 plays a crucial role in providing the desired consistency, viscosity, and stability. It forms a gel network by swelling in water, which entraps norfloxacin and other excipients, preventing their easy dispersion and ensuring uniform drug distribution within the gel matrix. The addition of carbopol-940 enhances the gel’s mechanical strength and improves drug retention, promoting controlled drug release over an extended period. The gel’s pseudoplastic flow behavior also facilitates smooth application, as it reduces resistance during spreading and improves patient compliance. Furthermore, carbopol-940’s ability to stabilize the gel formulation helps maintain the integrity of the product during storage and transportation. (c) Hydroxypropyl methyl cellulose (HPMC) is a hydrophilic polymer used as a gelling agent and viscosity modifier in pharmaceutical formulations. In the norfloxacin gel, HPMC complements the properties of poloxamer-407 and carbopol-940. It enhances the gel’s rheological properties, providing pseudoplastic flow behavior that aids in smooth application and ease of spreading. HPMC contributes to the gel’s bioadhesive properties, improving its adhesion to the skin or mucosal surfaces and increasing drug retention time at the application site. This prolonged contact promotes better drug absorption and therapeutic efficacy. Additionally, HPMC’s moisture-retaining properties maintain proper hydration of the gel, ensuring its stability and preventing gel shrinkage or cracking.

The norfloxacin gel was made by putting together poloxamer-407, carbopol-940, and HPMC in a way that takes advantage of their individual properties and creates a synergistic effect. The gelation of poloxamer-407 is triggered by temperature, and the thickening and stabilizing properties of carbopol-940 create an ideal gel matrix that allows controlled drug release and a longer time for the gel to stay in the ear where it is applied. HPMC complements this by enhancing the gel’s flow behavior and bioadhesion, providing better patient comfort and improved drug absorption during in situ gel application. This well-balanced formulation ensures the successful delivery of norfloxacin for effective treatment in otic applications [30].

### 4.3. Gel Formulation Preparation

**Figure 6:**
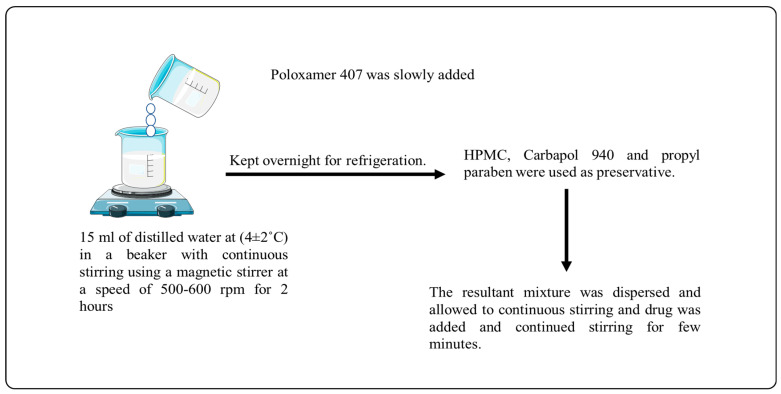
Method of preparation of Reverse gelation gel.

The gel was made using a cold technique. Poloxamer-407 was added to 15 mL of distilled water in a beaker with a magnetic stirrer at 500–600 rpm for 2 h at a temperature of 42 °C with continuous stirring(Figure 6). Refrigeration was carried out overnight (Figure 6). When the poloxamer dispersion was mixed with HPMC K-100 (0.5% *w/v*), carbopol-940 (0.1, 0.3%, and 0.5%), and propyl paraben (0.1%), the mixture was stirred constantly. Solubilizing the preservative in hot water yielded the preservative solution. After cooling, it was included in the aforesaid dispersion. Tween 80 and ethanol (1:2) were used to dissolve the weighed quantity of medication (2% *w/v*). Poloxamer dispersion was then added to the medication solution. Carbopol-containing dispersion was adjusted to pH 5.8 using triethanolamine, and the poloxamer-containing dispersion to pH 7 using triethanolamine. The compositions of the in situ gel formulations are shown in Table 9.

### 4.4. QbD Design

Design-Expert software, which DOE utilizes, was used to investigate the polynomial response surface and to create a polynomial model (Trial Version 13, Stat-Ease Inc., Minneapolis, USA). Using a full factorial design, we were able to statistically optimize the concentration of polaxamer-407 (gm) (X_1_), the concentration of carbapol 940 (gm) (X_2_) and the concentration of HPMC K100 (gm) (X_3_) as well as their interactions and the effects of the dependent variables percentage of entrapment efficiency (Y_1_), percentage of drug release (Y_2_) and viscosity (cps) (Y_3_). Table 10 illustrates the variables that are both coded and uncoded.

### 4.5. UV Spectroscopy and Calibration of Norfloxacin

#### 4.5.1. Scanning of Norfloxacin Aqueous Solution

The norfloxacin solution was made by dissolving 100 mg of norfloxacin in distilled water with 1 mL of glacial acetic acid and preparing a 1 mg/mL solution. A 100 mL volumetric flask has been used to make a stock solution of 1000 µg/mL. It has been determined that the diluted solution has a concentration of 100 µg/mL, and has been exposed to UV scanning at a wavelength of 200 to 400 nm.

#### 4.5.2. Calibration Curve of Norfloxacin Aqueous Solution

Methanol dilutions of 2, 4, 6, 8, and 10 µg/mL were created from a 1000 mg/mL stock solution before further testing. Methanol was used as a blank to measure the dilutions’ absorbance at 277 nm.

#### 4.5.3. Norfloxacin Phosphate Buffer Scanning and Calibration Curve (pH = 7.4)

Dissolving 100 mg of norfloxacin in 100 mL of phosphate buffer (pH 7.4) with 1 mg glacial acetic acid in a volumetric flask yielded a solution of 1 mg/mL norfloxacin. A confocal microscope was used to scan a 10 mL sample of 1000 µg/mL fluid in the 200–400 nm wavelength range. Diluting the stock solution to 1000 µg/ml at dilutions of 2, 4, 6, 8, and 10 µg/mL was done using phosphate buffer (pH = 7.5).

#### 4.5.4. Norfloxacin Scanning and Calibration Curve in Methanol

To make the stock solution and dilutions, 1 mL glacial acetic acid was dissolved in methanol [31].

### 4.6. Compatibility Study of Norfloxacin and Excipients

The IR spectra were recorded on a Shimadzu-8400 FTIR spectrometer using a KBr pellet technique. Dry KBr was compacted into pellets along with the addition of the drugs as well as the poloxamers 407 and HPMC, as well as propyl phenol and carbapol 940. To record the spectra, the pellets were placed in the sample holder.

### 4.7. Solubility Studies

The purpose of the solubility research was to find a solvent system in which the drug is readily soluble and to assess its solubility in a dissolving medium (pH 7.4 phosphate buffer). Glacial acetic acid, pure water, 7.4 phosphate buffer, 6.8 phosphate buffer, and methanol were all saturated with an excess of norfloxacin. Intermittent shaking was applied to the samples. To eliminate the undissolved drugs, the samples were filtered using Whatman filter paper (0.45 mm) after 24 h. Following that, appropriate dilutions were made, and norfloxacin was evaluated using a UV spectrophotometer at a maximum wavelength of 277 nm [32].

### 4.8. Partition Coefficient

Calculating the partition coefficient allows for the identification of the drug’s lipophilic/hydrophilic nature, which influences the rate of absorption. The partition coefficient (oil phase/aqueous phase) is a measurement of a drug’s lipophilicity and ability to cross cell membranes. Drugs with a log *p* value larger than 1 are categorized as lipophilic, whereas those with a partition coefficient less than 1 are classified as hydrophilic.

(4) $$P_{o/w}=\frac{C_{oil}}{C_{aq.}}$$

In a separating funnel, n-octanol was used as the oil phase and distilled water as the aqueous phase for the partition coefficient investigation. After two hours of vigorous shaking to get the combination to a state of equilibrium, it was left to stand overnight. The two phases were separated, and the absorbance at 0.417 nm and 0.275 nm of each phase was measured using a UV spectrophotometer (Table 11) [31].

### 4.9. Norfloxacin Reverse Gelatination Gel Evaluation

#### 4.9.1. Temperature of the Sol–Gel Transition

The temperature of the water bath was adjusted by 1 °C every five minutes from 33 to 40 °C for the sol–gel transition temperature test in many test tubes.

#### 4.9.2. Gelling Time

The gelling periods of the formulation were determined using a glass plate with the same slope as the ear, and the temperature was kept at 37 °C ± 0.5 °C. The gelling time was measured after the separate otic formulations (100–200 mL) were dropped on the glass plate. It was possible to see the transformation of a liquid solution into a thick gel. The -ve symbol is used to indicate preparations that did not gel. Those solutions with a phase transition after 90 s received the lowest score of +ve. The solutions that produced the gels in between 30 and 90 s received the maximum score of ++. The solutions that transitioned within 30 s and created stable gels for more than 30 min received the maximum score of +++.

#### 4.9.3. pH Measurement

Using a calibrated digital pH meter, the pH of each generated reverse gelatination gel formulation was recorded. The pH levels were measured both immediately after preparation and after 24 h of storage at room temperature [33].

#### 4.9.4. Rheological Investigation

The viscosity of norfloxacin reverse gelatination gel solution was measured using a Brookfield Viscometer at temperatures below 10 °C. The viscosity of the formulations was determined at 1–50 rpm at a temperature of 35–37 °C [34,35].

### 4.10. Zeta Potential

The zeta potential of the dispersion determines the stability of colloidal dispersion. The zeta potential value indicates the stability of the formulations [36].

### 4.11. Drug Content and % Entrapment Efficiency

**Figure 7:**
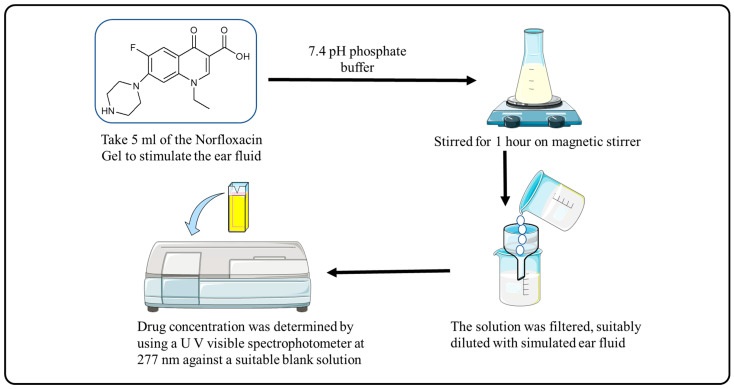
Procedure for Drug Content and % Entrapment Efficiency.

Adding 5 mL of the solution to stimulate the ear fluid with pH 7.4 phosphate buffer and stirring for 1 h on a magnetic stirrer was used to determine the drug content of norfloxacin-loaded reverse gelatination gel formulations. The solution was filtered and diluted with simulated ear fluid, and the drug concentration was evaluated against an appropriate blank solution using a UV visible spectrophotometer at 277 nm (Figure 7).

### 4.12. In-Vitro Release Kinetics

When selecting an appropriate system, the dissolution pattern is analyzed based on the model’s properties. In model-dependent approaches, the dissolution profile is characterized by a variety of mathematical functions [37,38]. The calculation of release kinetics is included in the supplementary files (S.3.1 to S.3.4).

### 4.13. Ex-Vivo Permeation Study

The ex-vivo permeation investigation was undertaken using the Franz diffusion cell. Porcine oral mucosa was used as a biological membrane in the investigation. Porcine oral mucosa was obtained from a slaughterhouse in the area and stored at 4 °C in phosphate buffer (pH 7) from the time of acquisition. Within three hours after purchasing it, it was put to use. The receptor compartment had phosphate buffer put into it (pH-7.4). This chamber also contains a stirring bead that is powered by a magnet. An appropriately sized membrane was placed between the donor and receptor compartments. A magnetic stirrer was used to keep the cell at 37 ± 1 °C while it was being swirled at 600 rpm. A gel sample of about 500 mg was placed in the donor compartment. Samples were taken at 15, 30, 45, 60, 75, 90, 120, 180, and 360 min. It was necessary to replenish the receptor compartment with an equal volume of fresh, hot phosphate buffer (pH 7.4) to maintain sink conditions. Before being evaluated for absorbance at 277 nm, the samples were filtered and diluted.

### 4.14. Stability Studies

The stability of the optimized formulation (F10) was evaluated over a period of 60 days at 4 °C and 25 °C. Parameters like pH, gelling capacity, and viscosity were evaluated at various time intervals. The pH of the formulation was measured using a pH meter. The gelling capacity of the formulation was assessed visually by observing whether it formed a gel when it was placed in the refrigerator. The viscosity of the sol and gel phases of the formulation was measured using a viscometer.

### 4.15. Network Pharmacology

The present analysis was undertaken in order to identify the exact mechanism of action on possible targets (STITCH (http://stitch.embl.de/ accessed, 9 May 2023), and GeneMania). Through the use of a Venn diagram, final targets have been identified.

## Figures and Tables

**Table 1:** Temperature of sol–gel transition in formulations F1–F10.

Sl. No	Formulation	Sol–Gel Transition Temperature	Gelling Time (s)
1	F_1_	35 ± 0.22 °C	90 ± 0.23
2	F_2_	34 ± 0.56 °C	120 ± 0.39
3	F_3_	35 ± 0.67 °C	80 ± 0.67
4	F_4_	35 ± 0.89 °C	50 ± 0.89
5	F_5_	36 ± 0.76 °C	90 ± 0.53
6	F_6_	34 ± 0.12 °C	35 ± 0.34
7	F_7_	36 ± 0.32 °C	80 ± 0.39
8	F_8_	35 ± 0.69 °C	90 ± 0.29
9	F_9_	34 ± 0.56 °C	58 ± 0.47
10	F_10_	33 ± 0.68 °C	40 ± 0.59

**Table 2:** Viscosity of formulations F1–F10 in solution and gel form.

Formulation	Viscosity (cp)			
	Solution State	Gel State	Spreadability	Gel Strength
F1	80.3 ± 1.14	1427.56 ± 0.39	5.46 ± 0.97	10.65 ± 0.67
F2	84.6 ± 0.17	1495.32 ± 0.43	4.34 ± 0.46	18.93 ± 0.53
F3	86.1 ± 0.56	1525.78 ± 0.89	5.12 ± 0.78	12.65 ± 0.36
F4	88.2 ± 0.45	1447.57 ± 0.65	4.57 ± 1.27	29.89 ± 0.85
F5	92.4 ± 0.34	1538.28 ± 0.56	4.93 ± 1.56	45.34 ± 0.47
F6	94.1 ± 0.67	1612.45 ± 0.74	5.27 ± 0.34	34.27 ± 1.32
F7	96.5 ± 0.39	1467.76 ± 1.05	4.5 ± 0.64	56.58 ± 1.48
F8	97.1 ± 0.69	1621.85 ± 3.68	5.67 ± 0.67	43.23 ± 0.45
F9	98.5 ± 0.78	1534.43 ± 1.14	5.29 ± 0.59	59.76 ± 0.37
F10	99.5 ± 1.45	1586.67 ± 1.14	6.35 ± 0.37	65.78 ± 0.45

**Table 3:** Shows the visual appearance and clarity of the pH.

Formulation	Visual Appearance	pH at 37 °C		
		At the Time of Preparation	After 24 h	After 48 h
F1	Solution in plain sight	7.23 ± 1.32	7.0 ± 2.43	7.0 ± 2.34
F2	Solution in plain sight	6.34 ± 2.56	6.22 ± 1.34	6.21 ± 1.34
F3	Solution in plain sight	7.45 ± 3.67	7.53 ± 4.34	7.52 ± 0.89
F4	Solution in plain sight	7.67 ± 2.89	7.0 ± 3.23	7.0 ± 0.56
F5	Solution in plain sight	7.49 ± 2.12	7.83 ± 1.23	7.84 ± 1.67
F6	Solution in plain sight	7.89 ± 3.56	7.29 ± 2.56	7.25 ± 2.89
F7	Solution in plain sight	7.6 ± 3.45	7.43 ± 2.67	7.46 ± 1.45
F8	Solution in plain sight	7.0 ± 2.67	7.12 ± 4.56	7.49 ± 4.34
F9	Solution in plain sight	6.57 ± 1.78	7.0 ± 2.34	7.0 ± 5.46
F10	Solution in plain sight	7.45 ± 1.23	7.47 ± 1.32	7.43 ± 6.45

**Table 4:** Kinetic analysis of in vitro release data of different formulations.

Formulations	Zero-Order		First-Order		Higuchi Model		Hixson–Crowell Model		Korsmeyer–Peppas Model		Best Fit Model
	K	r^2^	K	r^2^	K	r^2^	K	r^2^	K	r^2^	
F1	16.88	0.91	−0.25	0.904	48.69	0.98	−0.52	0.96	0.58	0.98	Higuchi model
F2	15.67	0.91	−0.20	0.911	45.21	0.98	−0.45	0.97	0.56	0.98	Peppas model
F3	19.33	0.92	−0.31	0.946	39.94	0.93	−0.56	0.93	0.54	0.97	Peppas model
F4	20.22	0.94	−0.31	0.937	42.09	0.94	−0.58	0.93	0.50	0.97	Peppas model
F5	20.66	0.90	−0.22	0.929	41.63	0.99	−0.45	0.98	0.72	0.98	Higuchi model
F6	23.38	0.94	−0.27	0.934	38.61	0.94	−0.59	0.96	0.73	0.95	Higuchi model
F7	15.46	0.91	−0.23	0.924	41.83	0.97	−0.42	0.97	0.76	0.97	Peppas model
F8	19.67	0.92	−0.29	0.917	43.39	0.98	−0.43	0.98	0.45	0.94	Higuchi model
F9	18.34	0.93	−0.23	0.923	41.56	0.96	−0.42	0.97	0.56	0.96	Higuchi model
F10	17.23	0.94	−0.37	0.945	47.62	0.99	−0.43	0.98	0.67	0.98	Higuchi model

**Table 5:** Drug contents of formulations F1–F10.

Formulation	% Drug Content	% Entrapment Efficiency
F1	80.58 ± 0.14	68.75 ± 0.28
F2	83.11 ± 0.65	55.98 ± 0.15
F3	80.92 ± 0.25	87.73 ± 0.50
F4	77.71 ± 0.44	59.07 ± 0.05
F5	83.82 ± 1.15	82.36 ± 0.36
F6	79.84 ± 0.48	55.78 ± 0.05
F7	78.70 ± 1.15	52.10 ± 0.47
F8	77.06 ± 0.30	84.18 ± 0.58
F9	75.18 ± 1.15	91.80 ± 0.23
F10	85.9 ± 0.26	95.78 ± 0.21

**Table 6:** Comparison of percentages of in vitro release of optimized formulation (F10) and pure drug.

Time	% CDR of Pure Norfloxacin	% CDR of Optimized Formulation of Norfloxacin
0.5	6.22222 ± 4.19	0.391 ± 8.39
1	8.54547 ± 0.04	5.149 ± 8.17 *
2	8.936111 ± 6.49	8.388 ± 1.41
3	11.10494 ± 3.10 **	17.149 ± 2.73
4	12.28364 ± 8.25	26.190 ± 3.21 **
5	14.62346 ± 0.87	37.405 ± 2.23
6	17.74753 ± 8.17 **	41.813 ± 8.56 *
7	31.20926 ± 5.74	46.747 ± 6.19
8	43.28395 ± 4.16	50.907 ± 6.39
9	46.1284 ± 7.87 **	57.558 ± 4.16 **
10	46.1284 ± 3.31	61.178 ± 4.06
11	49.86605 ± 5.64	68.906 ± 1.25 **
12	54.44228 ± 1.15	81.286 ± 1.45

* = Significant; ** = Highly Significant.

**Table 7:** Ex vivo release data of different formulations.

Time (min.)	%Release									
	F1	F2	F3	F4	F5	F6	F7	F8	F9	F10
15	25.209	18.203	22.569	24.223	25.203	24.453	23.203	17	15.584	18.234
30	35.645	32.549	34.457	32.254	30.3.45	35.109	26.940	24.625	27.125	22.963
45	49.480	45.904	43.554	42.765	47.489	43.489	38.404	37.766	41.667	34.839
60	65.467	63.266	60.687	64.543	57.616	60.526	58.126	57.880	59.337	56.71
75	74.303	72.079	71.224	76.205	69.177	70.778	67.212	68.800	66.151	77.45
90	87.642	85.993	86.343	83.231	80.776	78.923	82.007	76.876	78.470	71.405
120	96.648	97.729	95.709	91.709	89.708	90.129	94.120	87.685	84.825	79.856
180	90.392	95.234	91.105	87.773	94.343	92.221	89.103	88.065	93.132	85.009
360	97.845	96.325	99.715	94.413	98.705	99.098	97.775	90.642	91.654	95.653

**Table 8:** Stability studies on formulation F10.

Parameter	30 Days	45 Days	60 Days
pH	6.8	7	7.2
Gelling capacity	+++	+++	+++
Viscosity sol	15.1	15.3	14.6
Viscosity gel	99.5	99.5	97.1

+++ = Immediate gelation and leftovers for extended duration.

**Table 9:** Composition of formulations.

Formulations	Drug (gm)	T + E (mL)	P407 (gm)	C-940 (gm)	HPMC K-100 (gm)	PP (gm)	D/W (mL)
F1	0.54	6	4.5	0.1	0.05	0.004	30
F2	0.54	6	4.6	0.1	0.05	0.004	30
F3	0.54	6	4.7	0.1	0.05	0.004	30
F4	0.54	6	4.8	0.2	0.05	0.004	30
F5	0.54	6	4.9	0.2	0.05	0.004	30
F6	0.54	6	5.0	0.2	0.05	0.004	30
F7	0.54	6	5.1	0.3	0.05	0.004	30
F8	0.54	6	5.2	0.3	0.05	0.004	30
F9	0.54	6	5.3	0.3	0.05	0.004	30
F10	0.54	6	5.4	0.3	0.05	0.004	30

**Table 10:** Variables in factorial design.

Independent Variables	Level Used, Actual (Coded)		
	Low (−1)	Medium (0)	High (+1)
Independent variable X_1_ = Concentration of polaxamer-407 (gm)	4.5	5.0	5.4
X_2_ = Concentration of HPMC K100 (gm)	0.05	0.10	0.15
X_3_ = Concentration of carbapol 940 (gm)	0.1	0.2	0.3

Dependent variables Y_1_ = percentage of entrapment efficiency, Y_2_ = percentage of drug release, Y_3_ = viscosity (cps).

**Table 11:** Partition coefficient of norfloxacin.

Organic Solvent/Aqueous Phase	Absorbance (Oil Phase)	Absorbance (Aq. Phase)	*p* = C_1_/C_2_	Partition Coefficient (Log *p*)
n-Octanol/Distilled Water	0.419	0.274	1.521	0.182
	0.418	0.275		
	0.415	0.277		
Average Value	0.417	0.275		

## Data Availability

Not applicable.

## Supplementary Materials

The following supporting information can be downloaded at: https://www.mdpi.com/article/10.3390/gels9080657/s1, Figure S1: Calibration curve of (A) Norfloxacin scanning in methanol (B) Norfloxacin in distilled water (C) Norfloxacin in phosphate buffer at pH 7.4.; Figure S2: IR spectra of (A) Norfloxacin + Polaxamer 407; (B) Norfloxacin + Carbapol 940; (C) Norfloxacin + HPMC.; Figure S3: Drug Release kinetics Zero order, First order and Higuchi model.
