# Supplementary material for: QbD Design, Formulation, Optimization and Evaluation of Trans-Tympanic Reverse Gelatination Gel of Norfloxacin: Investigating Gene-Gene Interactions to Enhance Therapeutic Efficacy
Source: Gels. 2023 Aug 15;9(8):657. doi: 10.3390/gels9080657 (PMC10454480; doi:10.3390/gels9080657)
Supplement: Supplementary file 1 [file gels-09-00657-s001.zip › gels-2543034-supplementary.pdf]

Supplementary Results

S.1. UV Spectroscopy and Calibration of Norfloxacin

The UV absorption measurements of pure norfloxacin at 273 nm in phosphate buffer saline (PBS) showed linearity (r^2= 0.997) across the concentration range of 2-10 μg/mL, passing through the origin, and following Beer's law. The calibration curve between absorbance and concentration (μg/mL) was constructed and is shown in Figure 2. The slope and intercept values of Lambert's calibration curve were 0.108 and 0.009, respectively. The linearity of the UV absorption data at 273 nm and concentration estimation of pure norfloxacin across the concentration range of 2-10 μg/mL passing through the origin indicates that the drug follows Beer's law. This is an important finding, as it suggests that the drug can be accurately quantified using UV spectroscopy. The slope and intercept values of the calibration curve provide information about the Beer's law constant and the blank absorbance, respectively. The slope value of 0.108 indicates that the absorbance of norfloxacin increases linearly with increasing concentration. The intercept value of 0.009 indicates that there is a small amount of absorbance in the blank solution, which is likely due to the solvent or other components of the formulation. The linearity of the UV absorption data and the Beer's law constant suggest that UV spectroscopy is a reliable method for quantifying norfloxacin.

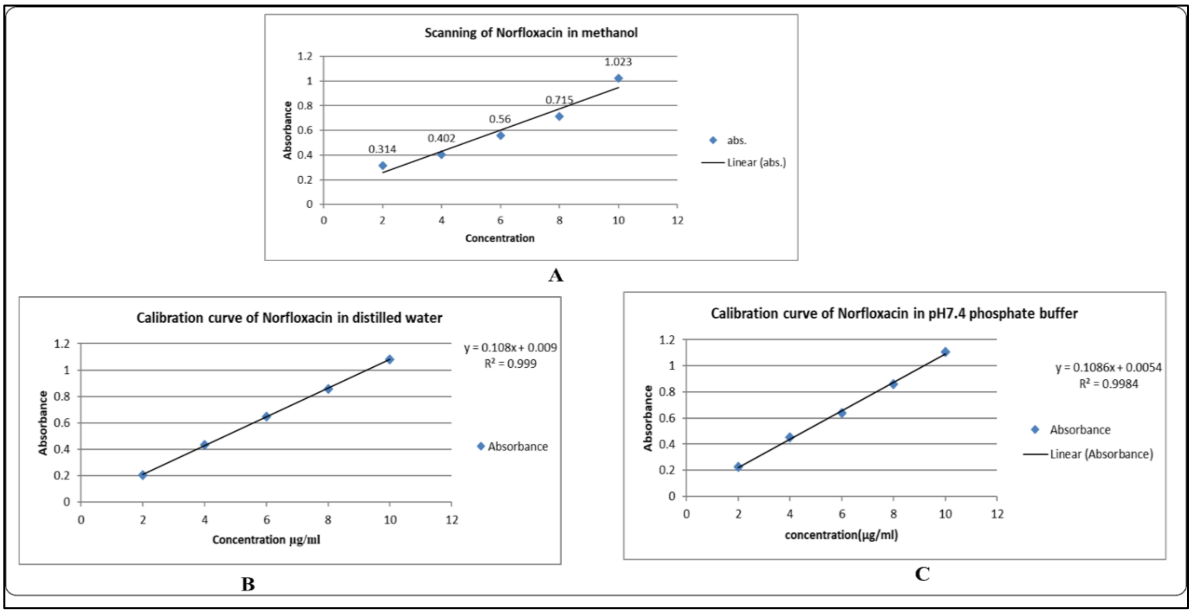

Figure S1. Calibration curve of (A) Norfloxacin scanning in methanol (B) Norfloxacin in distilled water (C) Norfloxacin in phosphate buffer at pH 7.4.

S.2. IRCompatibility of Norfloxacin with the excipients

This image depicts Norfloxacin with Polaxamer 407 and Carbapol 940 as well as Propyl perben and HPMC in Figure 3. The infrared (IR) spectrum of norfloxacin exhibited characteristic peaks at 3600–3250^{-1} (N–H and O–H stretching vibrations), 2500 cm^{-1} (hydrogen-bonded O–H stretching vibration), 1725–1700^{-1} (carboxylic acid C=O stretching vibration), and 1250 cm^{-1} (C–F and carboxylic C–O stretching vibrations). The presence of all of these peaks in the IR spectra of the formulations

indicates that the drug and excipients were compatible and that there was no change in the chemical structure of the drug. This is an important finding, as it suggests that the formulations are likely to be safe and effective.

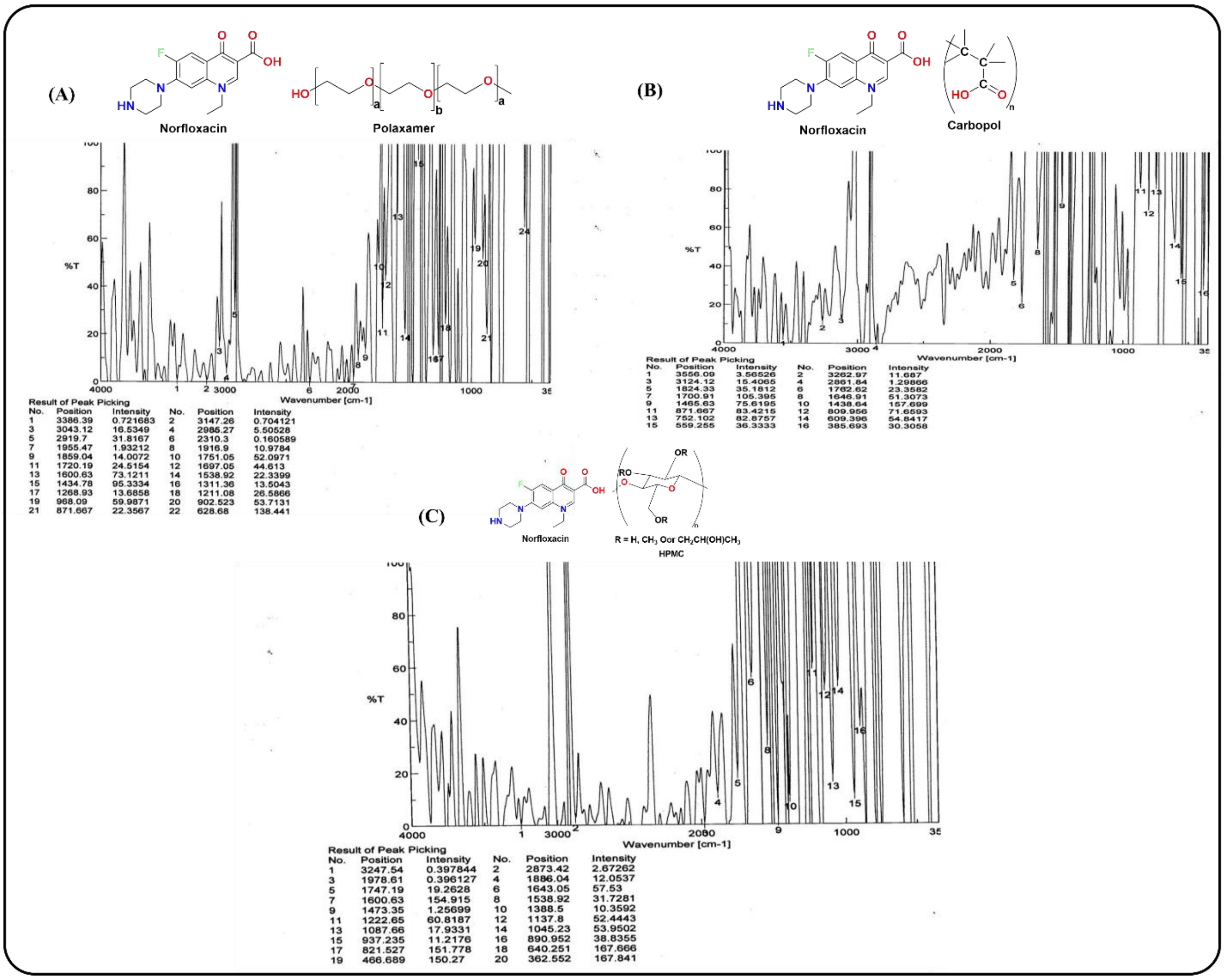

Figure S2. IR spectra of (A) Norfloxacin + Polaxamer 407; (B) Norfloxacin + Carbapol 940; (C) Norfloxacin + HPMC**.**

S.3.1. Kinetics of zero-order release

It establishes a straight line between the moment at which medication fractions are released and the fractions released.

$$Q = kt \quad (1)$$

Using this formula, we can calculate the proportion of the drug that has been released. For zero-order release, the plot of time-varying percentages of drug release is linear.

S.3.2. Kinetics of first-order release

First order release kinetics is described by the equation

$$In\ (1-Q) = -kt \quad (2)$$

Where Q is the percentage of medication released at time t and K is the first-order release rate constant. Thus, if the release kinetics are first

order, a logarithmic plot of the proportion of the drug remaining with time will be linear.

S.3.3. Higuchi equation

A plot of the proportion of drugs released vs the square root of time is linear if the release follows Higuchi's equation.

$$Q = kt^{1/2} \quad (3)$$

Where Quotient (Q) is the percentage of medication that has been released to this point in time (t).

S.3.4. Korsemeyer – Peppas kinetics

It is common to adopt the peppas model when the release mechanism is not well understood or many types of release are involved.

$$\text{Log } Q = \log k + n \log t \quad (4)$$

Where k is the release rate constant. It is common to adopt the peppas model when the release mechanism is not well understood or many types of release are involved. Mathematical equation:

$$M_t/M_\infty = kt_n \quad (5)$$

It is important to note that M_t/M_∞ is the ratio of drug released at time 't' as reflected by the diffusional exponent, n. There are two types of nonfickian release: one that is more than one and the one that is less than one. It has been shown that Peppas (1985) used the n value to distinguish between different release mechanisms, while studying zero-order release (case II transport) and super case II transport (n > 1).

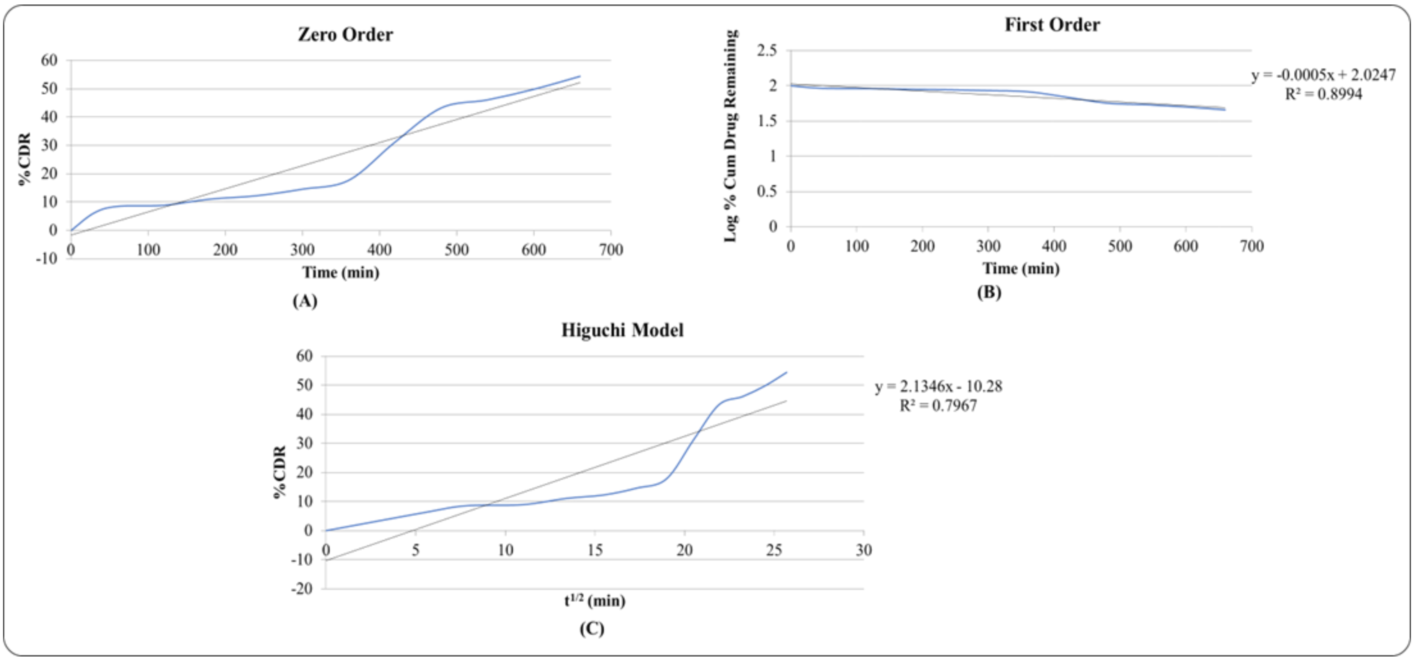

Figure S3. Drug Release kinetics Zero order, First order and Higuchi model.
